# Engaging in Physical Activity in Green Spaces at Night Is Associated with Mental Well-Being and Happiness

**DOI:** 10.3390/bs15030313

**Published:** 2025-03-05

**Authors:** Chun Jiang, Xing Zhang, Siyuan Feng, Hansen Li

**Affiliations:** 1 Department of Police Tactics, Chongqing Police College, Chongqing 401331, China; 2 Department of Physical Education and Sport, Faculty of Sport Sciences, University of Granada, 18071 Granada, Spain; 3 Laboratory of Genetics, University of Wisconsin-Madison, Madison, WI 53706, USA; 4 School of Physical Education, Sichuan Agricultural University, Yaan 625014, China

**Keywords:** exercise, nature, urban greening, psychological health

## Abstract

This study aims to explore the impact of the timing (day vs. night) and location (green space vs. non-green space) of outdoor physical activity on college students’ mental health. We designed a cross-sectional study based on self-reported data, asking participants to recall their physical activity and mental health status over the past month through a questionnaire. Specifically, a survey was conducted at a university in Chongqing, collecting data on outdoor physical activity and mental health indicators (including anxiety, depression, mental well-being, life satisfaction, happiness, and stress) from 418 students (75 females). The questionnaire was distributed via an online platform, allowing students to complete it using either their mobile phones or computers. The data collection took place in December 2024. The results showed that about half of the participants preferred engaging in outdoor physical activity in the nighttime, with most choosing green spaces. Regression analysis revealed that participants who engaged in outdoor physical activity at night had significantly lower anxiety levels compared to those who engaged in outdoor physical activity during the day (mean difference (MD) = −1.015; 95% CI = −1.974 to −0.055; *p* = 0.038). Additionally, compared to participants who engaged in outdoor physical activity in green spaces, those who engaged in physical activity in non-green spaces reported lower levels of mental well-being (MD = −1.531; 95% CI = −2.480 to −0.582; *p* = 0.002) and subjective happiness (MD = −0.462; 95% CI = −0.917 to −0.007; *p* = 0.047). Sensitivity analysis indicated that, for those who participated in nighttime activities, engaging in outdoor physical activity in green spaces was associated with higher levels of mental well-being (MD = 2.025; 95% CI = 0.810 to 0.324; *p* = 0.001) and happiness (MD = 0.583; 95% CI = 0.026 to 1.140; *p* = 0.040). Sensitivity analysis also revealed slight gender differences; however, the findings related to females should be interpreted with caution due to the insufficient sample size. Overall, despite some differences in time and location choices, engaging in outdoor physical activity at night in green spaces appears to associate with college students’ health, particularly their happiness and mental well-being. This study provides preliminary evidence of the potential benefits of nighttime green outdoor physical activity for improving college students’ mental health and offers directions for future research in this area.

## 1. Introduction

Green spaces are regarded as a bridge for human interaction with nature. A wealth of research has demonstrated the health benefits of exposure to green spaces. First, green spaces help alleviate stress, anxiety, and depression, thereby improving mental health (Z. Liu et al., 2023; Roe et al., 2013; van den Berg et al., 2010). Second, green environments encourage outdoor physical activities, reducing the risk of obesity and cardiovascular diseases (De la Fuente et al., 2021; X.-X. Liu et al., 2022). Furthermore, green spaces enhance social interactions, foster community cohesion, and increase overall well-being among residents (Jennings & Bamkole, 2019; Wan et al., 2021; X. Wang et al., 2023).

Similarly to nature exposure, physical activity can benefit human health, such as improving cardiovascular health, reducing mood disorders, and lowering all-cause mortality (Ahmed et al., 2012; Nocon et al., 2008; Saxena et al., 2005). Among these benefits, outdoor physical activity in natural environments is particularly deemed beneficial. Such activities are often referred to as “green physical activity” or “green exercise” (Pretty et al., 2003, 2005; Yeh et al., 2016). Green physical activity can be seen as a more advanced form of nature exposure (Pretty, 2004), combining the benefits of nature exposure and physical activity, which may be more conducive for health than physical activity alone (Shanahan et al., 2016).

Existing research has extensively explored the differential health benefits of physical activity in various environments (e.g., green vs. non-green environments) (de Brito et al., 2020; Kobayashi et al., 2018; Olafsdottir et al., 2018; Song et al., 2013). Meta-analyses have also provided positive evidence, indicating that engaging in green physical activity in natural environments yields improved emotional responses (Li et al., 2022b).

Given the numerous health benefits of green physical activity, scholars suggest that residents should engage in such activities. In this context, urban green spaces, which are public and open spaces with vegetation coverage (Schipperijn, 2010), provide an ideal setting for such activities. However, research on this topic remains relatively limited, particularly with regard to the temporal aspect of green physical activity. While many studies have examined the health benefits of green physical activity through controlled experiments, these studies have mostly focused on daytime activities, with nighttime physical activity receiving insufficient attention (Li et al., 2020).

With increasing societal competition and changing lifestyles, people’s activity durations have gradually extended, and many leisure activities, including outdoor physical activities, now often take place at night. This is particularly true in China, where long working and study hours are common (X. Liu et al., 2021; Zheng et al., 2023), potentially leading to insufficient physical activity. The shift toward nighttime activities may also pose challenges in terms of safety and accessibility, further affecting people’s ability to stay active. Therefore, the opportunity for physical activity during the nighttime warrants further attention.

In fact, existing research has underlined that although current physical activity guidelines provide recommendations on activity type, intensity, frequency, and duration, they do not address the timing of physical activity (Yi et al., 2022). Compared to daytime environments, nighttime environments differ significantly in terms of landscape and perceived safety (Bennett et al., 2007; Huang & Wang, 2018), and these differences may influence individuals’ physical activity behaviors and their associated health benefits. Therefore, for those who primarily engage in physical activity at night, whether they still choose green spaces for their activities and whether this choice remains beneficial for their mental health, is a question that has yet to be answered.

To fill this research gap, the present study aims to explore the time and location choices of outdoor physical activity among college students and further investigate the relationship between these choices and mental health indicators.

Our research hypotheses are as follows: (1) more people choose to engage in physical activity at night and in green spaces, and (2) the time and location of physical activity have interactive effects on mental health indicators.

## 2. Materials and Methods

### 2.1. Participants

This study employed a cross-sectional observational design and was conducted through a questionnaire survey at a university in Chongqing, where criminology is the primary major, in December 2024. All participants self-reported their physical activity and mental health status over the past month in a single survey session. The questionnaire was developed using an online survey platform provided by Wenjuanxing. Students received a link or QR code, which allowed them to complete the survey using either a computer or a mobile device. The inclusion criteria for participants were as follows: (1) university students; and (2) no severe physical disabilities preventing them from engaging in outdoor activities independently. The exclusion criterion was as follows: (1) failure to pass an attention test. In the first step of the survey, informed consent was obtained from the participants, and only those who volunteered participated in the study. The research process was supervised and approved by the Ethics Committee of Southwest University. After recruitment and screening, 418 participants were included.

### 2.2. Instruments

#### 2.2.1. Time and Location of Physical Activity

To assess the time and location of outdoor physical activity, the following two questions were used:

(1) “In the past month, when did you primarily engage in outdoor physical activity (such as walking, cycling, active exercise, etc.)?”

Options: “Daytime” and “Nighttime (e.g., after dark, when the local city lighting system begins operating)”.

(2) “In the past month, where did you primarily engage in the outdoor physical activities?”

Options: “Green space (e.g., parks, community or school green areas, etc.)” and “Non-green space (e.g., plazas, roads without vegetation, etc.)”.

Note that the “past month” time frame was chosen to avoid short-term fluctuations in physical activity behavior (e.g., students may significantly reduce outdoor activities in a given week due to weather conditions or academic commitments). Additionally, this time frame is commonly used in previous studies (Ainsworth et al., 2015; Brownson et al., 2005; Li et al., 2022a).

#### 2.2.2. Anxiety

The Generalized Anxiety Disorder-7 (GAD-7) in its Chinese version was used to assess participants’ anxiety levels (Sun et al., 2021). This scale is unidimensional and consists of 7 items, focusing on anxiety symptoms experienced over the past two weeks, such as excessive worry, difficulty relaxing, fatigue, and trouble concentrating. Each item was rated on a 4-point Likert scale (where 1 = not at all and 4 = nearly every day), and the total score was used to assess the level of anxiety.

#### 2.2.3. Depression

The Patient Health Questionnaire-9 (PHQ-9) in its Chinese version was used to assess participants’ depressive symptoms (W. Wang et al., 2014). This is also a unidimensional scale with 9 items, measuring depressive symptoms over the past two weeks, such as feelings of low mood, loss of interest, changes in appetite, and sleep issues. Each item was rated on a 4-point Likert scale (where 1 = not at all and 4 = nearly every day), with the total score reflecting the severity of depression.

#### 2.2.4. Mental Well-Being

The World Health Organization Five Well-Being Index (WHO-5) in its Chinese version was used to assess participants’ mental well-being (Fung et al., 2022). This short and effective scale measures overall well-being and psychological state. It consists of 5 items that assess individuals’ mood, mental state, and social interactions over the past two weeks. Each item was rated on a 6-point Likert scale, where 1 represents “at no time” and 6 represents “all the time”. The scores of each item were reversely coded after measurement, and the total score was used to indicate the level of mental well-being.

#### 2.2.5. Stress, Happiness, and Life Satisfaction

Due to the length constraints of the survey, we referred to previous studies and used single-item questions to assess participants’ perceived stress Littman et al., 2006), happiness (Abdel-Khalek, 2006), and life satisfaction (Cheung & Lucas, 2014) over the past week. Responses were measured using an 11-point Numerical Rating Scale (NRS), where 1 = “not at all” and 11 = “extremely”.

#### 2.2.6. Demographic Characteristics

We collected data on participants’ basic demographic characteristics, including sex, age, monthly family income, and academic year.

### 2.3. Analysis

#### 2.3.1. Correlation

Given the different types of our variables, we employed different methods to calculate the correlations. For the psychological scale measurements, which are positive integers and ordinal, we used Spearman’s correlation to detect the relationship between two variables. The time and location of physical activity were dichotomous, so we used Phi correlation to measure their relationship. Additionally, for the associations between the time or location of physical activity and psychological variables, we used point-biserial correlation, treating the time or location as dichotomous variables and the psychological variables as continuous.

#### 2.3.2. Regression

To explore the effect of the location and time of physical activity on mental health, we conducted regression analysis using generalized linear models (GLMs). For mental well-being, happiness, and stress, we used basic linear models with normal distribution and identity link. For anxiety and depression, because the data were notably right-skewed, we applied models with gamma distribution and logarithmic links (Ng & Cribbie, 2017). The gamma-based model demonstrated lower AIC values compared to linear models, confirming its appropriateness.

In the regression models, all health-related outcome variables measured by the scales were treated as continuous variables, as is consistent with the approach of similar studies (Dzhambov et al., 2018). To avoid multicollinearity issues, we computed the variance inflation factor (VIF) for all variables, including the interaction term for physical activity location and time, ensuring that all VIF values were below 5.0 (Shrestha, 2020). Additionally, we included demographic variables (gender, age, family income, academic year, etc.) in the models to control for confounding factors. If the interaction effects were detected, we further conducted simple effects analysis. If the main effects of the two dichotomous variables (time or location of physical activity) were significant, we performed post hoc pairwise comparisons after regression, and presented the mean differences between groups, along with the corresponding 95% confidence intervals and significance levels.

In addition, for variables that showed significant differences between different times or locations, we also included Cohen’s d as an additional parameter to help reflect effect sizes (Luo et al., 2022). A Cohen’s d of 0.2 is considered a small effect size, while 0.5 and 0.8 are considered medium and large effect sizes, respectively (Luo et al., 2022).

#### 2.3.3. Sensitivity Analysis

Following the STROBE guidelines (Cuschieri, 2019), we also performed sensitivity analysis. Specifically, we stratified the data by the time of physical activity (daytime vs. nighttime) and used a general linear regression model with HC3 robust standard errors to investigate the relationship between physical activity location and mental health outcomes. The robust standard errors help mitigate the impact of heteroscedasticity. Furthermore, when the sample size was sufficient (e.g., the sample-to-parameter ratio exceeded 10), the OLS-based regression model showed a high tolerance for residual distribution (Schmidt & Finan, 2018) Moreover, we stratified the main analysis by gender to examine the differences between males and females. In this study, a *p*-value less than 0.05 was considered statistically significant. All data analysis was conducted using SPSS version 26.

## 3. Results

### 3.1. Participant Characteristics

Due to the academic nature of the university, the majority of participants (82.1%) in this study were male, with an average age of approximately 20 years (Table 1). Most participants had a monthly family income between CNY 5001 and 10,000 (as of 12 January 2025, 1 CNY ≈ 0.14 USD). The majority of participants (53.3%) were third-year students. Regarding outdoor physical activity, over 70% of participants reported that they primarily engaged in activities in green spaces. As for the time of activity, the proportion of participants who chose daytime (47.6%) or nighttime (52.4%) activities was roughly equal.

### 3.2. Correlation Analysis

The correlation analysis revealed that participants who engaged in outdoor physical activity in green spaces were more likely to do so during the daytime (Phi = 0.141, *p* = 0.004). In addition, compared to those who engaged in outdoor physical activity in green spaces, participants who engaged in physical activity in non-green spaces reported lower levels of happiness (r_pb_ = −0.122, *p* = 0.013) and mental well-being (r_pb_ = −0.174, *p* < 0.001). Furthermore, compared to participants who primarily engaged in physical activity during the daytime, those who engaged in outdoor physical activity at night exhibited lower levels of anxiety (r_pb_ = −0.097, *p* = 0.048) and depression (r_pb_ = −0.097, *p* = 0.047), but also reported lower happiness levels (r_pb_ = −1.115, *p* = 0.019) (Table 2).

### 3.3. The Effects of Time and Location on Mental Health Variables

We did not observe any significant interaction effects between the location and time of physical activity (Table 3). However, compared to participants who engaged in physical activity primarily during the daytime, participants who engaged in outdoor physical activity at night had significantly lower anxiety levels (mean difference (MD) = −1.015; 95% CI = −1.974 to −0.055; *p* = 0.038; Cohen’ d = 0.193). Additionally, compared to participants who engaged in outdoor physical activity in green spaces, those who engaged in physical activity in non-green spaces reported lower levels of mental well-being (MD = −1.531; 95% CI = −2.480 to −0.582; *p* = 0.002; Cohen’ d = 0.379) and happiness (MD = −0.462; 95% CI = −0.917 to −0.007; *p* = 0.047; Cohen’ d = 0.276).

### 3.4. Sensitivity Analysis

In the stratified analysis by physical activity time, two significant associations were identified (Table 4). First, for participants who primarily engaged in outdoor physical activity at night, those who chose green spaces over non-green spaces had significantly higher mental well-being (MD = 2.025; 95% CI = 0.810 to 0.324; *p* = 0.001; Cohen’ d = 0.411) and happiness levels (MD = 0.583; 95% CI = 0.026 to 1.140; *p* = 0.040; Cohen’ d = 0.284).

In the gender-stratified analysis, we found that the results for males were generally consistent with the main findings (Table 5). However, for females, the location showed a significant main effect on life satisfaction and happiness. Specifically, females who engaged in physical activity in green spaces reported higher levels of happiness (MD = 1.524, 95% CI = 0.226 to 2.823; *p* = 0.021; Cohen’ d = 0.747) and life satisfaction (MD = 1.467, 95% CI = 0.306 to 2.627; *p* = 0.013; Cohen’ d = 0.848).

## 4. Discussion

This study, based on a questionnaire survey, aims to explore the interactive effects of outdoor physical activity location and timing on mental health. We used a convenience sampling method to select a sample of students from a university, predominantly male. This sample differs from the general Chinese population, so the findings have relatively low external validity. Nevertheless, this study provides preliminary insights into this topic and offers reference points for strategies to improve the health of university students.

Regarding the specific results, we found that 72.2% of our participants reported primarily engaging in physical activity in green spaces. Meanwhile, 52.4% of our participants reported primarily engaging in physical activity at night. These results partially support our first hypothesis (more people choose to engage in physical activity at night and in green spaces). We did not observe any significant interaction effects between the location and time of physical activity, which does not support our second hypothesis (that the time and location of physical activity have interactive effects on mental health indicators).

Nevertheless, we found that, compared to participants who primarily engaged in physical activity during the daytime, participants who engaged in outdoor physical activity at night had significantly lower anxiety levels. Additionally, compared to participants who engaged in outdoor physical activity in green spaces, those who engaged in physical activity in non-green spaces reported lower levels of mental well-being and happiness. It is worth noting that the effect sizes observed in our main analysis were small to moderate. Therefore, these associations should not be exaggerated.

In our sensitivity analysis, we found that among participants who primarily engaged in outdoor physical activity at night, those who chose green spaces over non-green spaces had significantly higher mental well-being and happiness levels. Furthermore, when stratified by gender, we found that the results for males were generally consistent with the main findings. In females, the location showed a significant main effect on life satisfaction and happiness. Specifically, females who engaged in physical activity in green spaces reported higher levels of happiness and life satisfaction.

Overall, there is some inconsistency between the results of the main analysis and the sensitivity analysis, which is understandable. First, the interaction effect analysis assumed that both the timing and location of physical activity directly impact mental health, whereas the stratified regression primarily examined the moderating effect of outdoor physical activity timing on the strength of the relationship between “activity location” and “mental health”. In other words, the sensitivity analysis did not consider the potential effects of activity timing on mental health outcomes.

Despite these differences, the results from both analyses collectively suggest that outdoor physical activity in green spaces at night is associated with enhanced mental well-being and happiness.

### 4.1. Timing and Location of Outdoor Physical Activity

Regarding location preferences, many psychological theories highlight the appeal of natural environments. For instance, the biophilia hypothesis suggests that humans have an innate biological and genetic connection with nature, which affects not only physiological but also emotional aspects, potentially influencing human behavior (Gaekwad et al., 2022). This theory can explain people’s intrinsic motivation to engage with nature. For our participants, an innate preference for nature may increase their likelihood of engaging in behaviors that bring them closer to natural environments. This process of engaging with nature is often primarily manifested through physical activities, such as walking, which helps explain their choices of locations for physical activity. Furthermore, other psychological theories, such as the Stress Recovery Theory, Attention Restoration Theory, and Environmental Adaptation Theory, all emphasize that exposure to natural environments contributes to positive psychological experiences (Li & Zhang, 2023), providing theoretical support for choosing green spaces for physical activity.

A recent qualitative study also reported that participants mentioned the comfortable characteristics of green spaces as one of the reasons they chose to exercise at night (Su et al., 2024), further supporting the role of green spaces in promoting nighttime physical activity.

### 4.2. The Relationship Between Nighttime Outdoor Physical Activity and Mental Health

The results of this study collectively suggest that outdoor physical activity in green spaces during the night is positively associated with happiness and mental well-being. Over the past decade, numerous experimental studies have documented the positive effects of engaging in green physical activities on mental health (Coventry et al., 2021; Li et al., 2022b). Observational studies have also identified beneficial associations among green physical activity and mental well-being, anxiety, and depression (Li et al., 2022a, 2023). While we observed a positive association between engaging in physical activity in green spaces and mental health, we did not find significant relationships with anxiety and depression. This discrepancy may arise from the different methodologies used in prior studies, which typically focused on quantifiable parameters of green physical activities, such as frequency and duration (Li et al., 2022a, 2023), whereas our study only focused on the binary choice of outdoor activity location. Moreover, although not related to green exercise specifically, some studies have shown a positive relationship between exposure to green spaces and happiness (Patino et al., 2023; Soga et al., 2021). These results align with our findings and emphasize the benefits of being close to green spaces.

It is important to note that the aforementioned evidence was not obtained specifically in the context of “nighttime”. Based on our main and sensitivity analyses, we may conclude that engaging in outdoor physical activity in green spaces at night is beneficial for mental health. This finding is interesting, as daytime activities theoretically should provide a greater sense of safety and be more beneficial to health. We speculate that there may be several reasons for this. First, concerns about crime at night may be minimal due to good social security, particularly on university campuses. Second, considering the typical schedules of university students, nighttime physical activity may be more closely associated with leisure and recreation, which could lead to better relaxation. Finally, the nighttime environment is often quieter and more secluded, offering students more privacy and less environmental disturbance, particularly in green spaces. Currently, research specifically focusing on nighttime green physical activity is still limited. One previous study presented a preliminary comparison of emotional experiences during physical activity in urban environments and green spaces at different times of the day (Li et al., 2020). However, the study found no significant difference in emotional experiences between green spaces and non-green environments at night. Due to significant differences in the sample, research design, and focus of the study, these results are not directly comparable to those of our study. Given the limited number of relevant studies, it is difficult to discuss our findings in greater depth.

Nevertheless, we wish to emphasize that despite the potential influence of safety concerns and other factors on nighttime green space use, nighttime green spaces may still provide a valuable venue for physical activity and play a positive role in promoting mental health. This underscores the need for further research in this area. Future studies should focus more on the specific impacts of nighttime green physical activity on mental health and its underlying mechanisms.

Finally, we would like to emphasize that, although it was not the main focus of our study, we observed some gender differences. For females, rather than males, engaging in physical activity in green spaces was associated with higher levels of happiness and life satisfaction. This highlights the impact of gender differences. Previous studies have shown that gender influences psychological traits and social roles, which may, in turn, affect the perception and use of green spaces, as well as the potential health benefits (Fernández Núñez et al., 2022a, 2022b; Sillman et al., 2022). However, due to the small sample size of females in our study, these findings should be interpreted with caution.

### 4.3. Limitations

Firstly, the cross-sectional design of this study limits our ability to draw causal inferences. While cross-sectional data can identify associations among time and location choices and mental health, it cannot determine whether these factors directly cause changes in mental well-being. A longitudinal study following participants over time would allow for a more robust understanding of causal relationships and help uncover the long-term effects of these contextual factors on mental health. For instance, such a study could reveal whether consistent patterns in time and location choices lead to sustained improvements or declines in mental health over time, or if other variables mediate these effects. In addition, other methods, such as ecological analysis based on instrumental variables, could help clarify the causal direction of these associations. This is worth exploring if the dataset allows.

Secondly, the sample population was limited to university students, which introduces potential sample bias. While this group is an important demographic, particularly in studying the mental health of young adults, their unique lifestyle and stressors may not reflect those of other age groups, cultural backgrounds, or professional environments. University students often face specific pressures related to academic performance, social relationships, and transitions to adulthood, which may differ from the experiences of individuals in the workforce or other life stages. Therefore, the generalizability of the findings to broader populations is limited. Future studies should aim to include more diverse populations, such as young professionals, older adults, or people from different cultural contexts, to determine how time and location choices impact mental health across a range of life stages and environments.

Moreover, while the participants in this study were from a university primarily offering criminology programs, we did not account for the academic major as a potential confounder. Different academic disciplines may shape students’ schedules, time management strategies, and even their mental health outcomes in unique ways. For example, students in highly demanding majors may experience different levels of stress, social engagement, or physical activity compared to those in less demanding programs. By not controlling for academic major, we risk introducing confounding variables that could affect the interpretation of our results. Future research should incorporate the academic major as a key variable to explore whether its influence moderates the relationship between time/location choices and mental health outcomes.

Additionally, this study focused on the location and time of activity but did not delve into other important parameters such as the intensity or duration of physical activity, which are well-documented as significant factors influencing mental health (Bernstein et al., 2019; Kerr & Kuk, 2001). It is possible that the impact of time and location choices varies significantly depending on how long and how intensely the activity is performed. For example, an activity that occurs in a peaceful environment but lasts only a short time may have different effects than prolonged activity performed in a more stressful location. Future research should examine how these parameters interact with time and location choices to provide a more nuanced understanding of their combined influence on mental well-being.

In conclusion, while this study has several limitations, its value lies in offering a novel framework for future investigations into the relationship among physical activity, mental health, and contextual factors. By addressing these limitations—through longitudinal designs, more diverse samples, and inclusion of additional control variables—future research can build on these findings to provide deeper insights into the complex interactions at play. This study serves as a foundational step in understanding how the broader context of time and place affects mental well-being, paving the way for more comprehensive studies in the future.

## 5. Conclusions

This study explored the impact of time and location choices for outdoor physical activity on the mental health of university students, finding that nighttime physical activity in green spaces is associated with lower anxiety levels and higher happiness. Although no interaction effect between time and location was observed, some results suggest that the combination of nighttime and green spaces may offer unique benefits for mental health. Future research should further explore causal relationships and consider other characteristics of physical activity, as well as individual differences, to validate and expand upon these findings.

## Figures and Tables

**Table 1:** Participant demographics.

Variable	Category	N (%)	Mean (SD)
Sex	Male	343 (82.1%)	-
	Female	75 (17.9%)	-
Age	-	-	20.26 (1.22)
Family Income (monthly)	0–5000 CNY	101 (24.2%)	-
	5001–10,000	131(31.3%)	-
	10,001–15,000	103 (24.6%)	-
	15,001–20,000	42 (10.0%)	-
	20,001–25,000	21 (5.0%)	-
	25,001–30,000	3 (0.7%)	-
	>30,000 CNY	17 (4.1%)	-
Academic Year	Freshman	47 (11.2%)	-
	Sophomore	120 (28.7%)	-
	Junior	223 (53.3%)	-
	Senior	19 (4.5%)	-
	Graduate	9 (2.2%)	-
Physical Activity Location	Green Space	302 (72.2%)	-
	Non-Green Space	116 (27.8%)	-
Physical Activity Time	Daytime	199 (47.6%)	-
	Nighttime	219 (52.4%)	-
Anxiety (GAD-7)	-	-	7.22 (4.92)
Depression (PHQ-9)	-	-	3.92 (5.89)
Life Satisfaction	-	-	7.01 (1.97)
Happiness	-	-	7.12 (2.07)
Stress	-	-	5.31 (2.4)
Mental Well-being (WHO-5)			17.05 (4.31)

**Table 2:** Correlations between key variables.

Variable	1	2	3	4	5	6	7
**1. PA Location**	1						
**2. PA Time**	0.141 **	1					
**3. Anxiety**	0.050	−0.097 *	1				
**4. Depression**	0.024	−0.097 *	0.804 **	1			
**5. Mental Well-being**	−0.174 **	−0.037	−0.431 **	−0.449 **	1		
**6. Life Satisfaction**	−0.089	−0.049	−0.342 **	−0.370 **	0.482 **	1	
**7. Happiness**	−0.122 *	−0.115 *	−0.393 **	−0.407 **	0.496 **	0.849 **	1
**8. Stress**	−0.061	−0.074	0.259 **	0.197 **	−0.187 **	−0.058	−0.120 *

**Note:** *, *p* < 0.05; **, *p* < 0.01. Activity location is coded as “Green space” = 0 (reference group) and “Non-green space” = 1; activity time is coded as “Daytime” = 0 (reference group) and “Nighttime” = 1. PA, physical activity.

**Table 3:** The effects of outdoor physical activity time and location on mental health.

Dependent Variable	Independent Variable	Wald Chi-Square	df	Significance
Anxiety	Location	2.457	1	0.117
	Time	4.374	1	0.036
	Location × Time	0.150	1	0.699
Depression	Location	0.780	1	0.377
	Time	2.948	1	0.086
	Location × Time	0.932	1	0.334
Mental Well-being	Location	10.013	1	0.002
	Time	0.506	1	0.477
	Location × Time	0.925	1	0.336
Life Satisfaction	Location	2.146	1	0.143
	Time	0.560	1	0.454
	Location × Time	0.124	1	0.725
Happiness	Location	3.953	1	0.047
	Time	3.756	1	0.053
	Location × Time	0.331	1	0.565
Stress	Location	1.14	1	0.286
	Time	0.445	1	0.505
	Location × Time	1.187	1	0.276

**Note:** All analyses were adjusted for age, gender, family income, and academic year.

**Table 4:** The effects of physical activity location on mental health.

Variable	Time	Location	MD	*p*	95% CI	
					Lower	Upper
Anxiety	Daytime	Green space	−0.453	0.619	−2.244	1.339
	Daytime	Non-green space	Ref.	-	-	-
	Nighttime	Green space	−0.951	0.124	−2.167	0.264
	Nighttime	Non-green space	Ref.	-	-	-
Depression	Daytime	Green space	0.148	0.883	−1.839	2.135
	Daytime	Non-green space	Ref.	-	-	-
	Nighttime	Green space	−1.049	0.141	−2.449	0.352
	Nighttime	Non-green space	Ref.	-	-	-
Mental Well-being	Daytime	Green space	0.852	0.353	−0.954	2.657
	Daytime	Non-green space	Ref.	-	-	-
	Nighttime	Green space	2.025	0.001	0.810	3.240
	Nighttime	Non-green space	Ref.	-	-	-
Life Satisfaction	Daytime	Green space	0.180	0.620	−0.535	0.895
	Daytime	Non-green space	Ref.	-	-	-
	Nighttime	Green space	0.421	0.112	−0.099	0.941
	Nighttime	Non-green space	Ref.	-	-	-
Happiness	Daytime	Green space	0.280	0.493	−0.523	1.083
	Daytime	Non-green space	Ref.	-	-	-
	Nighttime	Green space	0.583	0.040	0.026	1.140
	Nighttime	Non-green space	Ref.	-	-	-
Stress	Daytime	Green space	0.639	0.170	−0.276	1.553
	Daytime	Non-green space	Ref.	-	-	-
	Nighttime	Green space	0.031	0.917	−0.562	0.625
	Nighttime	Non-green space	Ref.	-	-	-

**Note:** All analyses were adjusted for age, gender, family income, and academic year.

**Table 5:** Main results stratified by gender.

		Male			Female		
	Independent Variable	Wald Chi-Square	df	*p*	Wald Chi-Square	df	*p*
Anxiety	Location	1.643	1	0.200	0.312	1	0.577
	Time	4.709	1	0.030	0.723	1	0.395
	Location × Time	0.608	1	0.435	0.824	1	0.364
Depression	Location	0.051	1	0.820	2.392	1	0.122
	Time	2.764	1	0.096	1.322	1	0.250
	Location × Time	1.619	1	0.203	0.615	1	0.433
Mental Well-being	Location	8.556	1	0.003	0.717	1	0.397
	Time	1.174	1	0.279	2.498	1	0.114
	Location × Time	1.987	1	0.159	2.497	1	0.114
Life Satisfaction	Location	0.222	1	0.637	6.135	1	0.013
	Time	0.802	1	0.371	0.207	1	0.649
	Location × Time	0.514	1	0.474	0.570	1	0.450
Happiness	Location	0.912	1	0.340	5.293	1	0.021
	Time	5.172	1	0.023	0.331	1	0.565
	Location × Time	0.684	1	0.408	0.541	1	0.462
Stress	Location	2.397	1	0.122	0.357	1	0.550
	Time	0.076	1	0.783	1.863	1	0.172
	Location × Time	1.747	1	0.186	0.414	1	0.520

**Note:** All analyses were adjusted for age, family income, and academic year.

## Data Availability

Data will be available upon request to the corresponding author.
